# HIV‐associated nephropathy: A decade of clinical outcomes, global disparities and emerging promise

**DOI:** 10.1111/hiv.70315

**Published:** 2026-09-29

**Authors:** Deepak V. Pathiyil, Nicola Wearne, Brendon Price, Frank Post, Dami A. Collier

**Affiliations:** ^1^ Department of Medicine University of Cambridge Cambridge UK; ^2^ Cambridge University Hospitals NHS Foundation Trust Cambridge UK; ^3^ University of Cape Town Cape Town South Africa; ^4^ National Health Laboratory Service (NHLS), University of Cape Town Cape Town South Africa; ^5^ Kings College London London UK; ^6^ Kings College Hospital NHS Foundation Trust London UK; ^7^ Department of Pathology University of Cambridge Cambridge UK

**Keywords:** antiretroviral therapy, *APOL1*, chronic kidney disease, collapsing focal segmental glomerulosclerosis, health disparities, HIV‐associated nephropathy, kidney transplantation, sub‐Saharan Africa

## Abstract

**Objective:**

To review the pathogenesis, epidemiology, clinical presentation, management and outcomes of HIV‐associated nephropathy (HIVAN), with emphasis on global disparities, genetic susceptibility (*APOL1*) and emerging therapeutic and technological advances.

**Design:**

Narrative literature review synthesizing contemporary evidence from clinical cohorts, translational research and guideline‐based practice, with comparative perspectives from high‐resource (UK) and low‐resource (sub‐Saharan Africa) settings.

**Methods:**

Relevant studies were analysed to evaluate mechanisms of disease, epidemiological trends, diagnostic approaches, treatment strategies and long‐term outcomes. Particular focus was placed on *APOL1*‐associated risk, antiretroviral therapy (ART) impact and recent developments including artificial intelligence (AI) applications and targeted therapies.

**Results:**

HIVAN arises from direct HIV infection of renal epithelial cells in genetically susceptible individuals, particularly those with *APOL1* risk variants. ART has significantly reduced incidence in high‐income settings, whereas HIVAN remains prevalent in sub‐Saharan Africa due to delayed diagnosis and limited healthcare access. Clinically, HIVAN presents with proteinuria and progressive kidney dysfunction, requiring biopsy for confirmation. Early ART initiation improves renal outcomes, supported by adjunctive therapies such as renin‐angiotensin system blockade and SGLT2 inhibitors. Emerging therapies targeting *APOL1* and advances in AI‐driven risk prediction and diagnostics show promise.

**Conclusions:**

HIVAN reflects an interplay between viral, genetic and socio‐economic factors. While ART has transformed outcomes in well‐resourced settings, significant global disparities persist. Future progress depends on improving HIV care access, expanding renal services and advancing precision medicine approaches, including *APOL1*‐targeted therapies and AI‐enabled risk stratification.

## INTRODUCTION

HIV‐associated nephropathy (HIVAN) is a collapsing form of focal segmental glomerulosclerosis (FSGS) to which people of African ancestry with HIV are at a greatly increased risk. First recognized in the 1980s as a rapidly progressive form of kidney failure in individuals with advanced immunodeficiency, HIVAN remains an important cause of kidney disease in people of African ancestry living with HIV [1].

Over the past two decades, widespread use of combination antiretroviral therapy (ART) has altered the landscape of HIVAN, reducing its incidence in regions with good HIV care coverage while the disease persists in areas with limited resources. Importantly, HIV‐related kidney diseases encompass a broad spectrum. According to the Kidney Disease: Improving Global Outcomes (KDIGO) consensus classification [2], these fall into four major pathological categories: (i) Glomerular‐dominant nephropathies – including classical HIVAN (collapsing FSGS) and immune‐mediated complications of HIV, (ii) Tubulointerstitial‐dominant nephropathies – including HIVAN‐related tubulointerstitial injury, ART toxicity (e.g. tenofovir), other drug‐induced interstitial nephritis and infections such as tuberculosis (TB) affecting the kidney, (iii) Vascular‐dominant nephropathies – such as thrombotic microangiopathy (now rare in the ART era), and (iv) Other nephropathies in the setting of HIV – notably comorbid diseases like diabetic or hypertensive nephropathy in an ageing population [1, 2].

This narrative review provides a comprehensive overview of the pathogenesis, epidemiology, diagnosis, treatment and clinical outcomes of HIVAN, with a particular focus on progression to kidney failure, recovery of kidney function and long‐term prognosis. Special emphasis is placed on observations from sub‐Saharan Africa and the United Kingdom, illustrating global disparities in HIVAN burden and outcomes.

## PATHOGENESIS OF HIVAN

HIVAN is characterized by the direct infection of kidney epithelial cells by HIV, leading to a cascade of cellular injury and dysfunction in susceptible individuals. The virus predominantly targets glomerular podocytes and tubular epithelial cells, despite these cells lacking the conventional HIV entry receptors (CD4, CCR5, CXCR4) [3]. Research in the last decade has shed light on alternative mechanisms of viral entry: for example, pro‐inflammatory signals such as transmembrane TNF‐α can ‘prime’ kidney cells for HIV infection by facilitating viral entry and integration [4] and direct cell‐to‐cell transfer via tunnelling nanotubules may allow HIV to infect kidney cells without free virus in the extracellular space [5].

Once inside the kidney, HIV triggers a profound dysregulation of normal cell cycle controls. Podocytes, which are normally terminally differentiated, begin to proliferate (evidenced by high Ki‐67 expression) and dedifferentiate, losing key structural proteins like synaptopodin [6]. This results in the classic collapsing glomerular lesions of HIVAN: the capillary tufts collapse and overlying epithelial cells may form pseudocrescents, accompanied by microcystic dilation of tubules filled with proteinaceous casts (i.e. microcysts). Tubulointerstitial inflammation and fibrosis are also prominent, potentially involving pathways such as TGF‐β/SMAD3 and notch signalling that drive apoptosis and scarring [7].

A pivotal discovery in understanding HIVAN pathogenesis has been the role of host genetic factors, particularly variants in the *apolipoprotein L1* (*APOL1*) gene. *APOL1* risk alleles (the G1 and G2 variants, common in individuals of sub‐Saharan African ancestry due to past selective pressure from African trypanosomiasis) confer a dramatically increased susceptibility to HIVAN. Possessing two *APOL1* risk variants raises the odds of developing HIVAN by roughly 29‐fold in African Americans [8], and studies of Africans in South Africa and the United Kingdom have reported similar or even higher odds ratios (up to ~89‐fold) for HIVAN in those with high‐risk *APOL1* genotypes [9, 10].

However, not everyone with a high‐risk genotype develops kidney disease – it is estimated that two *APOL1* risk alleles carry a 50% lifetime risk of nephropathy in the context of untreated HIV infection [8]. This suggests that a ‘second hit’ is required in addition to genetic susceptibility [11, 12]. In people with HIV, HIV infection itself may serve as a trigger: viral proteins and interferon‐driven inflammation can upregulate *APOL1* expression in kidney cells, pushing susceptible podocytes into injury pathways [13].

Recent reviews and transcriptomic analyses have clarified the pathogenic mechanisms of variant *APOL1* on podocytes. The expression of risk variants appears to cause a toxic gain‐of‐function. Key mechanisms identified include: (i) Mitochondrial dysfunction, where *APOL1* variants impair mitochondrial respiration and bioenergetics, leading to metabolic reprogramming and cell death [14], (ii) Endolysosomal trafficking defects, causing swelling of lysosomes, and (iii) Ribosomal stress, resulting in downregulation of ribosomal proteins and translational arrest in *APOL1* high‐risk cells [15], (iv) Additionally, the insertion of *APOL1* variants into the cell membrane forms pH‐gated cation channels that facilitate the efflux of potassium and influx of sodium, triggering stress kinases and cytotoxicity – a mechanism that is the direct target of novel therapies. APOL1 risk variant is neither necessary nor sufficient to cause HIVAN. This is supported by the occurrence of HIVAN in approximately 20% of individuals with a single APOL1 risk allele, and occasionally among individuals with wild‐type APOL1. This highlights the incomplete penetrance of APOL1‐associated risk and supports the requirement for additional viral, inflammatory or host ‘second hits’ in disease pathogenesis [10].

## EPIDEMIOLOGY AND GLOBAL DISTRIBUTION

In the ART era, the epidemiology of HIVAN has shifted markedly. People with HIV overall remain at elevated risk of kidney disease (both HIVAN and other nephropathies) compared to HIV‐negative peers. Chronic kidney disease (CKD) affects an estimated ~6%–8% of adults with HIV globally, with the highest prevalence in regions of sub‐Saharan Africa (~7–8%) [16].

HIVAN, the classic HIV‐related CKD, has become less common in well‐resourced settings with near‐universal ART access; simultaneously, other kidney diseases (such as hypertensive nephrosclerosis, diabetic nephropathy and immune complex glomerulonephritides) have become more prevalent as people with HIV live longer. For example, a 10‐year renal biopsy series from Sydney, Australia reported no cases of HIVAN, a finding attributed to the low prevalence of African ancestry in that population [11]; comparably low HIVAN frequencies have been described in other well‐resourced cohorts in which few people of African or Caribbean ancestry are represented. By contrast, in parts of Africa where many people still present with advanced, untreated HIV, HIVAN remains a leading cause of kidney pathology [17].

It is critical to interpret prevalence estimates with caution due to survival bias. HIVAN is an aggressive disease that can progress to kidney failure in weeks; in settings with limited access to renal replacement therapy (RRT), individuals often succumb to uraemia or complications before they can be captured in cross‐sectional studies [18]. Consequently, prevalence surveys likely underestimate the true burden of the disease. Furthermore, while South Africa has produced robust biopsy data, there is a notable paucity of clinical cohort studies characterizing HIVAN in West Africa, despite this region being the epicentre of *APOL1* genetic diversity.

As shown in Figure 1, the distribution of *APOL1* variants follows a distinct gradient: high‐risk genotypes are most prevalent in West Africa (correlated with *Trypanosoma brucei gambiense* endemicity) and persist in Southern Africa but are virtually absent in East Africa [12, 19]. This genetic epidemiology aligns with clinical observations where HIVAN is rare in East African populations despite high HIV burden.

**FIGURE 1:**
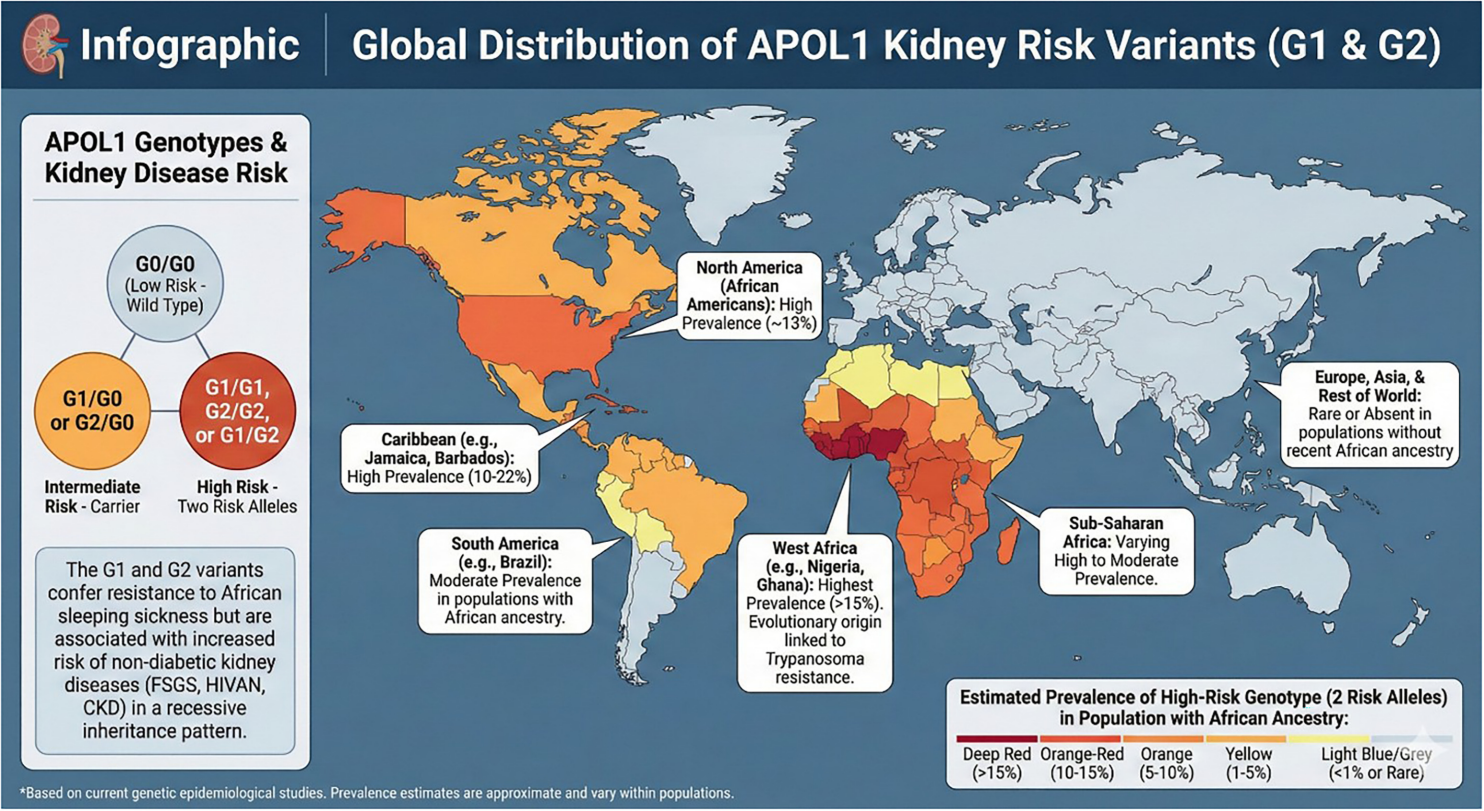
Global Distribution of APOL1 Kidney Risk Variants. The map highlights the West‐to‐East gradient of APOL1 risk variants across Africa, with the highest prevalence in West Africa (e.g. Nigeria, Ghana) and near absence in East Africa (e.g. Ethiopia). [12, 19].

Sub‐Saharan Africa bears a disproportionate share of both the HIV pandemic and HIVAN cases. With an estimated 25.6 million people living with HIV in the region, late presentation is common and ART coverage, while improving, is suboptimal and heterogeneous. [20]. The introduction of ART has begun to change this picture: centres that rolled out ART earlier saw significant declines in the proportion of kidney biopsies showing HIVAN. A recently published 15‐year biopsy series from Cape Town, South Africa confirms this trend. In that cohort (2005–2020), the prevalence of HIVAN as a biopsy diagnosis fell from ~45% in the mid‐2000s to ~29% by the late 2010s, coincident with expanding ART access [21]. Over the same interval, the frequency of tubulointerstitial diseases (often related to HIV/Tb co‐infection or tenofovir toxicity) rose from ~13% to 33% of biopsies [21].

In the United Kingdom and other high‐resource settings, new‐onset HIVAN has become an uncommon diagnosis over the past ten years, thanks to early HIV detection and universally available treatment. By 2023, over 107 000 people were accessing HIV care in the United Kingdom [22]. Large UK studies have shown that HIVAN is almost exclusively confined to people of Black African or Caribbean ancestry. A seminal UK cohort study by Post et al. identified 61 HIVAN cases among 16 834 people with HIV, with no cases in white patients [23]. All 61 were of Black African or Caribbean origin, and notably 83% of them had HIVAN diagnosed concurrently with their first positive HIV test [23]. The GEN‐AFRICA study (2018–2020) further highlighted within‐group differences in the UK's African diaspora: West African ancestry was associated with over six‐fold higher risk of biopsy‐proven HIVAN/FSGS compared with East or Southern African ancestry among Black people with HIV [10].

## CLINICAL PRESENTATION AND DIAGNOSIS

Individuals with HIVAN typically present with proteinuria, often nephrotic‐range proteinuria with minimal peripheral oedema, normal or low blood pressure and enlarged echogenic kidneys on ultrasound; urinalysis is usually bland (no active sediments) [24]. The median estimated GFR at diagnosis is often ~15–30 mL/min in many biopsy‐proven series, reflecting late presentation [24]. HIVAN strongly correlates with advanced HIV disease: CD4 counts tend to be low (often <200 cells/μL, and in many cases <50) and HIV viral loads high at the time of kidney disease onset [24].

Definitive diagnosis of HIVAN requires a kidney biopsy, as clinical criteria alone are imprecise (positive predictive value only ~55%–60% in some series) [25]. The classical kidney biopsy in HIVAN shows the pathognomonic pattern of collapsing FSGS: segmental or global collapse of glomerular capillary tufts with overlying podocyte hypertrophy and hyperplasia forming pseudocrescents. Tubular segments show microcystic dilation filled with proteinaceous casts, and there is often marked interstitial inflammation and fibrosis. Structurally, endothelial tubuloreticular inclusions are frequently seen on electron microscopy, reflecting chronic interferon exposure [25].

It is important to distinguish HIVAN from other kidney diseases occurring in people living with HIV. In particular, immune complex‐mediated glomerular diseases in the setting of HIV infection, characterized by immune complex deposition on biopsy (e.g. IgG‐ and/or C3‐dominant deposits, including lupus‐like patterns), represent a heterogeneous group of entities. In contrast to HIVAN, these lesions are generally associated with a more indolent clinical course and a lower risk of progression to kidney failure [26, 27]. Nonetheless, an overlap can occur, and some biopsies can show mixed features. Given the therapeutic implications, most guidelines advocate for biopsy confirmation of HIVAN when feasible.

## TREATMENT AND MANAGEMENT

### Antiretroviral Therapy

Prompt initiation of fully suppressive ART is the cornerstone of HIVAN management. Achieving virological control limits ongoing kidney injury, and cohort studies have shown that kidney function (eGFR and proteinuria) stabilizes or improves in a large proportion of people after 6–12 months of ART, especially if started before advanced fibrosis has developed [28]. As people with HIVAN often present with severe systemic illness, it is possible that the improvement relates to resolution of acute tubular injury (ATI). Ongoing viraemia, on the other hand, predicts relentless decline in kidney function. Consequently, current HIV guidelines recommend starting ART as soon as HIVAN is diagnosed (regardless of CD4 count), if not already on therapy.

When selecting ART regimens in people with HIVAN or CKD, it is important to consider drug nephrotoxicity. Tenofovir disoproxil fumarate (TDF) can cause proximal tubular damage and is best avoided in those with impaired kidney function (tenofovir alafenamide, TAF, is a safer alternative in those with reduced GFR) [29]. Integrase strand transfer inhibitors (INSTIs) like dolutegravir, which are commonly used in individuals with CKD, can cause modest rises in serum creatinine by inhibiting tubular creatinine secretion. This represents a laboratory phenomenon that reflects reduced tubular secretion of creatinine rather than true nephrotoxicity or a genuine fall in glomerular filtration; it is non‐progressive, typically appears within the first weeks of therapy, and is not a contraindication to the use of INSTIs [30]. In a broader renal context – proteinuric kidney disease is usually managed with ACE inhibitors or ARBs, and SGLT2 inhibitors irrespective of diagnosis [27].

### Renin‐Angiotensin System Inhibition

ACE inhibitors or ARBs reduce intraglomerular pressure and proteinuria and may slow progression of FSGS lesions. Small studies and clinical experience suggest they provide renoprotection in HIVAN, so they are generally recommended (if tolerated) as adjunct therapy [31].

### SGLT2 Inhibitors

Sodium‐glucose cotransporter‐2 (SGLT2) inhibitors have emerged as a prominent tool in the management of proteinuric CKD. Originally developed for diabetes, landmark trials such as DAPA‐CKD and EMPA‐KIDNEY have demonstrated that SGLT2 inhibitors significantly reduce the risk of kidney disease progression and cardiovascular events in patients with CKD, independent of diabetes status [32, 33]. People living with HIV were largely excluded from these landmark trials, and so HIVAN‐specific randomized evidence is lacking. Dedicated evidence in this population is, however, limited but emerging rather than absent: a US cohort of people with HIV found SGLT2 inhibitors to be used with adverse event rates comparable to other glucose‐lowering agents [34], and a US prospective multi‐site study reported that, although initiation was associated with a modest and transient early dip in eGFR, longer term kidney function trajectories were consistent with those observed in HIV‐negative populations [35]. A further small cohort of people living with HIV, the majority of whom had CKD, documented favourable metabolic, immune and tolerability outcomes with SGLT2 inhibitors [36]. Taken together, these data support a position of limited but emerging evidence, while adequately powered HIVAN‐specific trials remain needed. Reflecting the consistency of benefit across diverse non‐diabetic CKD aetiologies and this reassuring early experience, both international and national guidance now support SGLT2 inhibitors for eligible patients with proteinuric CKD, including those with HIV. The KDIGO 2024 Clinical Practice Guideline recommends an SGLT2 inhibitor for adults with CKD and clinically significant albuminuria irrespective of diabetes status [37], and UK guidance similarly recommends SGLT2 inhibitors (e.g. dapagliflozin) for people with proteinuric CKD (urine albumin‐to‐creatinine ratio >25 mg/mmol) on optimized RAS blockade, including people with HIV, provided eGFR is adequate [38]. These agents reduce glomerular hyperfiltration and may mitigate the podocyte stress central to HIVAN pathophysiology [39].

### Corticosteroids

The role of corticosteroids in HIVAN remains unclear. In a single‐centre randomized study from South Africa, the addition of corticosteroids to ART was associated with a significant improvement in eGFR; a trend towards higher mortality was also observed in the corticosteroid group, but this did not reach statistical significance (*p* = 0.07) and may reflect the small number of events [40]. Steroids carry substantial risks in the context of HIV (opportunistic infections, poor glycaemic control, etc.), especially if viraemia is uncontrolled. On balance, corticosteroids are not routinely recommended in HIVAN outside specific indications and should be used with caution.

### Renal Replacement Therapy

Dialysis and transplantation are effective replacement options for people with HIVAN who progress to kidney failure. In settings where resources allow, dialysis outcomes in people with HIV can approach those of HIV‐negative patients, provided HIV is well‐controlled. In many low‐resource settings across sub‐Saharan Africa, access to chronic dialysis remains limited, and people living with HIV were historically excluded from renal replacement programmes. With the expansion of kidney transplantation, HIV infection is no longer considered a contraindication to dialysis or transplantation in several African countries, including South Africa. Kidney transplantation confers a substantial survival benefit, and multiple cohorts have demonstrated comparable 1‐ and 5‐year graft survival among HIV‐positive and HIV‐negative kidney transplant recipients. [41, 42]. Notably, South Africa also pioneered HIV‐positive‐to‐HIV‐positive kidney transplants with success [43, 44]. Historically post‐transplant, drug interactions between ART and immunosuppressants (e.g. protease inhibitors with calcineurin inhibitors) required careful management at a time when protease inhibitors were commonly used [45]. However, this is no longer an issue with the use of integrase inhibitors, and prophylaxis for opportunistic infections is continued lifelong. Overall, with appropriate patient selection and management, transplantation offers excellent outcomes and is now standard care for kidney failure in people with HIV in many settings.

## CLINICAL OUTCOMES AND PROGNOSIS

In the pre‐ART era, HIVAN often progressed to kidney failure within weeks to a few months from diagnosis [46]. In the current era, outcomes have improved dramatically with ART [47].

Long‐term outcomes now vary greatly by setting. In the United Kingdom and similar high‐resource environments, the rarity of new HIVAN cases means most patients now face CKD rather than acute kidney failure, managed with standard renoprotection and ART. Conversely, in sub‐Saharan Africa, while ART scale‐up has improved survival, late presentation remains a barrier. One‐year dialysis mortality in HIV‐positive African patients remains higher than in resource‐rich settings, and HIVAN still contributes substantially to kidney failure burdens where ART coverage lags [20].

Importantly, histopathological characteristics can predict prognosis. One South African study found that the presence of microcystic tubular dilatation on biopsy was a strong adverse prognostic indicator: individuals with tubular microcysts had a significantly higher risk of progression to kidney failure or death (adjusted hazard ~2.0) compared to those without [48]. Conversely, the use of ART at the time of biopsy was protective, reducing the risk of death or kidney failure by nearly half [49].

## HEALTHCARE DISPARITIES IN HIVAN

HIVAN sits at the intersection of viral illness, genetic susceptibility and social determinants of health.

- Racial/Genetic Disparities: The heavy concentration of *APOL1* risk alleles in populations of recent African ancestry explains much of the racial disparity. Approximately 90% of HIVAN‐kidney failure cases in the United States occur in African Americans [50]. In Africa, even within Black populations, those of West African heritage (where *APOL1* risk variants are most prevalent) have higher rates of HIVAN than those of East African heritage [10]. This genetic risk factor underlies the near absence of HIVAN in non‐African descent populations and those from the horn of Africa, despite similar HIV parameters.
- HIV Care Access: Late HIV diagnosis and gaps in treatment have historically sustained the burden of HIVAN in sub‐Saharan Africa, whereas aggressive outreach, routine testing and immediate ART initiation have greatly reduced HIVAN incidence in high‐income settings where implemented. In the United Kingdom and other high‐income countries, many HIVAN cases occur in migrants or persons with a late HIV diagnosis or who disengaged from care – reflecting that, globally, HIVAN often signals a failure of healthcare access. Strengthening HIV care delivery is a critical disparity‐mitigating strategy.
- Resource Limitations in Kidney Care: Dialysis and transplant access remain limited in much of sub‐Saharan Africa, necessitating rationing that contrasts sharply with high‐resource nations. Expanding nephrology infrastructure and training in low‐income settings is essential to close this gap.

## FUTURE DIRECTIONS IN HIVAN: AI AND APOL1‐TARGETED THERAPIES

Looking ahead, two promising fronts are emerging: the application of artificial intelligence (AI) in care and novel therapies targeting the *APOL1* pathway.

### AI and Predictive Modelling

AI tools are poised to improve prediction, diagnosis and management. Machine learning algorithms can leverage large HIV cohort datasets to predict CKD risk with high accuracy [51]. Such predictive models could be used to identify which individuals with HIV are at highest risk for kidney function loss (due to factors like uncontrolled viraemia, diabetes and potential genetic risks), prompting earlier interventions or closer monitoring of those individuals. It is conceivable that in the near future, an AI system could help detect subtle collapsing lesions or microcysts on biopsy scans, or even non‐invasively identify an HIVAN‐like injury pattern through urine biomarkers and clinical data integration. Additionally, ‘multi‐omics’ approaches (combining genomics, transcriptomics, proteomics) with AI may uncover new biomarkers that distinguish HIVAN from other kidney diseases or predict who will respond to therapies [52]. A concrete realization of this precision‐medicine approach has recently emerged: Li et al. derived and validated an APOL1 Proteomic Risk Score (APRS), a nine‐protein plasma signature that identifies APOL1 high‐risk individuals who will progress to kidney disease before overt CKD becomes apparent. The APRS achieved a time‐dependent AUC of approximately 86%, substantially outperforming the Kidney Failure Risk Equation (~66%) and polygenic risk scores, and retained robust accuracy on external validation [53]. Such data‐driven biomarkers move the field from aspiration towards deployable tools for risk stratification in APOL1‐associated disease, of which HIVAN is an example.

### *APOL1*‐Targeted Therapies

Because *APOL1* genetic variants are central to HIVAN pathogenesis, therapies counteracting these variants are in development. Two strategies have moved to the forefront: APOL1 inhibitors and JAK–STAT pathway blockers (Figure 2).

- Inaxaplin (VX‐147): This is a first‐in‐class small molecule *APOL1* inhibitor designed to block the *APOL1* channel. In a Phase 2a trial, participants achieved a ~47% reduction in proteinuria [54]. It is currently in Phase 2b/3 trials.
- MZE829: Another oral small‐molecule APOL1 inhibitor in clinical development. A Phase 2 trial (HORIZON) is underway to assess its effects on proteinuria and other outcomes in patients with APOL1 kidney disease.
- AZD2373: Additional investigational strategies include antisense oligonucleotides targeting APOL1 mRNA (e.g. AZD2373) that have shown Phase I activity and may progress towards Phase II development.
- JAK–STAT Inhibitors: As detailed in the pathogenesis section, high interferon states upregulate *APOL1* via the JAK–STAT pathway. Baricitinib, a JAK1/2 inhibitor, has been shown to block *APOL1* induction and rescue podocytes in preclinical models [55]. The JUSTICE trial, a phase 2 study of baricitinib in patients with *APOL1*‐mediated kidney disease [56] has completed in April 2026; results are awaited and will help determine whether JAK inhibition translates into clinical benefit in this setting.

**FIGURE 2:**
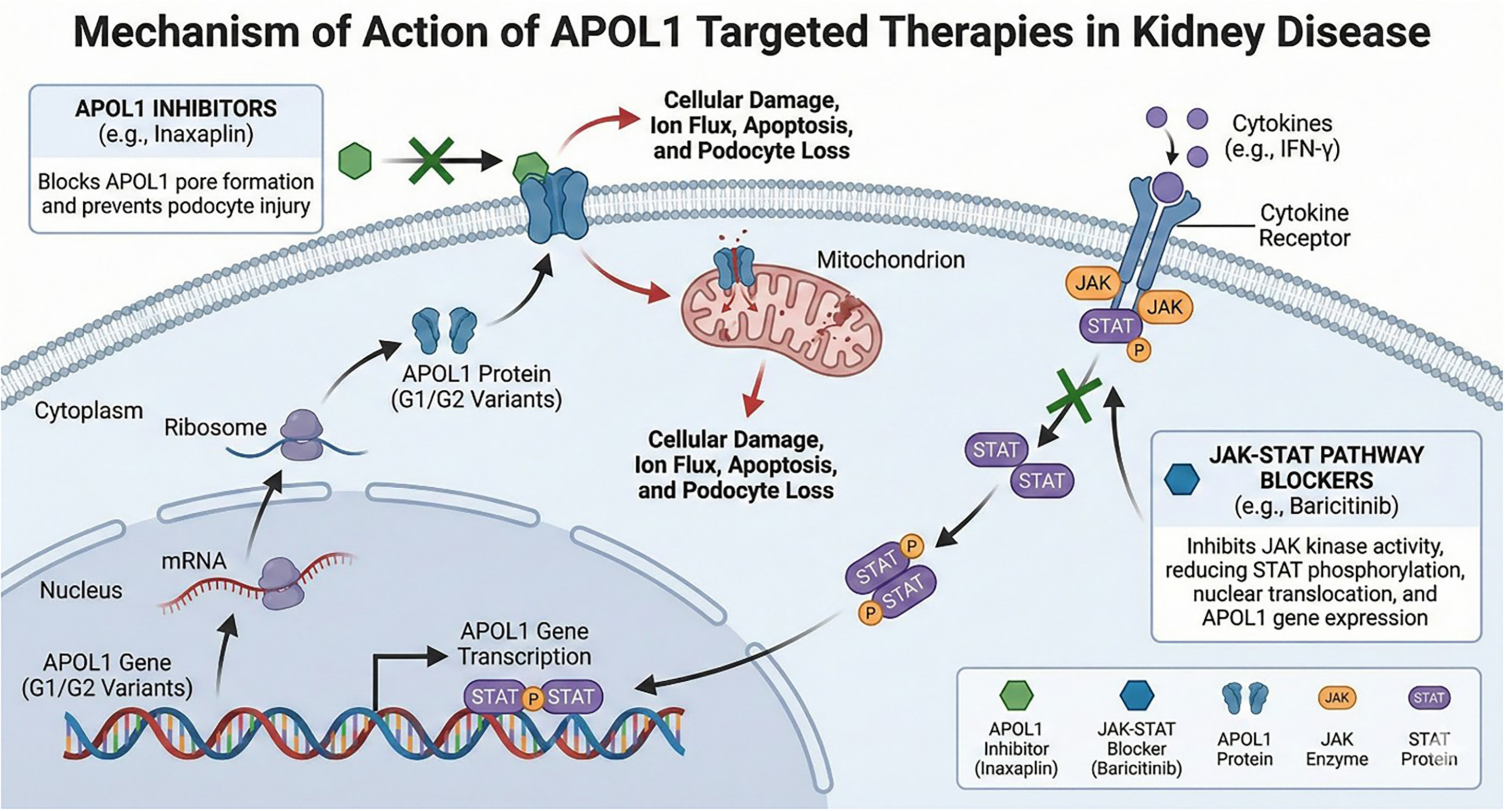
Mechanism of Action of APOL1 Targeted Therapies in Kidney Disease. The diagram illustrates the ‘toxic gain of function’ where APOL1 variants form cation channels in the podocyte membrane, leading to K+ efflux and cell injury. This pore formation is blocked by inhibitors like Inaxaplin. Downstream, cytokine signalling (JAK–STAT) upregulates APOL1 transcription, a process inhibited by drugs like Baricitinib. [13, 54, 55, 56].

Other strategies in earlier stages include gene‐silencing approaches to reduce *APOL1* expression [57].

## CONCLUSION

In summary, HIVAN arises from a convergence of viral factors (local HIV replication and/or inflammatory signals in the kidney) and host factors (genetic predisposition and immune dysregulation). This dual mechanism explains why HIVAN is almost exclusive to individuals of African descent with uncontrolled viraemia and underscores the importance of both virologic control and genetic context in the pathogenesis and management of HIVAN. Notably, the past 10 years have also seen experimental therapies targeting APOL1, though these remain in early stages of development.

Our understanding and management of HIVAN has greatly advanced over the last decade. Key to this progress has been the scale‐up of ART which, by controlling HIV viraemia, protects the kidneys from the deleterious effects of variant *APOL1*. We now recognize HIVAN as a disease arising from a synergy of direct HIV kidney infection, genetic susceptibility and social determinants.

Where HIV is well‐controlled, HIVAN has strikingly waned; where late presentation persists, HIVAN remains. Ongoing efforts must focus on closing care gaps through HIV prevention, early diagnosis and universal ART access. Adjunct therapies such as RAAS blockade and SGLT2 inhibitors may slow progression, and access to transplantation allows many to survive kidney failure. Looking forward, *APOL1*‐targeted drugs herald a new era of precision medicine for this condition and AI for risk stratification and prognostication.

## AUTHOR CONTRIBUTIONS

Deepak V Pathiyil (DVP) conceived the review, designed its scope, conducted the literature search and appraisal of previously published work in this field and drafted the manuscript. Nicola Wearne (NW), Brendon Price (BP), Frank Post (FP) and Dami A Collier (DAC) contributed to the conception and design of the review, contributed clinical, histopathological and epidemiological expertise and previously published work on this topic, identified and interpreted relevant literature and critically revised successive drafts. DAC supervised the work as senior author. All authors reviewed and approved the final version and agree to be accountable for the work.

## Data Availability

Data sharing not applicable to this article as no datasets were generated or analysed during the current study.
